# Gallbladder cancer presenting with a single giant common bile duct stone in a patient with normal cholestatic function tests: a case report

**DOI:** 10.1093/omcr/omag173

**Published:** 2026-09-14

**Authors:** Yitagesu Aberra Shibiru, Shimelis Nigussie, Amanuel Yegnanew, Dawit Dagne Moges

**Affiliations:** Department of Surgery, Adama Hospital Medical College, Adama, 192, Ethiopia; Department of Surgery, Addis Ababa University, Addis Ababa, Tikur Anbesa, Ethiopia; Department of Surgery, Addis Ababa University, Addis Ababa, Tikur Anbesa, Ethiopia; Department of Radiology, Kidus Petros Specialized Hospital, Addis Ababa, Ethiopia

**Keywords:** gallbladder cancer, CBD stone, giant CBD stone, dilated CBD

## Abstract

We present the case of a 65-year-old Ethiopian female with a 10.5 × 4 cm, 150 g giant CBD stone and normal cholestatic function tests. Ultrasound and contrast-enhanced CT scan suggested gallbladder cancer and a CBD stone with dilated intrahepatic and extrahepatic biliary trees. The patient was successfully managed with extended cholecystectomy, common bile duct exploration, stone extraction, and choledochoduodenostomy. Histopathology revealed adenocarcinoma of the gallbladder with negative margins. No tumor recurrence was noted on subsequent follow-up of 2 years. Giant CBD stones are rare, especially in Africa. Most patients present with abnormal cholestatic function tests and jaundice; however, presentation with normal cholestatic parameters is uncommon. The coexistence of gallbladder cancer and a giant CBD stone represents longstanding gallstone disease and chronic inflammatory changes that contribute to the development of gallbladder cancer.

## Background

Although there is geographic variation in the prevalence of gallbladder cancer (GBC), it is the sixth most common malignancy and the leading cancer of the biliary tract in the United States [1]. Geographic differences in possible risk factors are thought to contribute to variations in prevalence among different regions [2].

Risk factors for the development of gallbladder cancer include gallstones, obesity, female sex, Hispanic and Native American ethnicity, infections (HBV and HCV), primary sclerosing cholangitis, choledochal cysts, and anomalous pancreaticobiliary junction (APBJ), among others [3]. Around 80%–90% of patients with gallbladder cancer are reported to have gallstones [2]. Gallstones are a well-established risk factor, likely due to chronic inflammation, bacterial colonization, and recurrent mechanical injury.

The prevalence of gallstones varies among populations and is primarily influenced by age, ethnicity, gender, and genetics. According to the NIH (1993), 10%–15% of the population in the United States had gallstones [4]. Among patients with gallstones, the risk of developing GBC is less than 0.5% over 20 years [5]. Patients with gallstones have a 6%–10% risk of having an associated CBD stone, and the incidence increases with age. The vast majority of CBD stones are secondary (originating from the gallbladder), while primary stones are more common in the eastern part of the world [6].

Patients with common bile duct stones typically present with biliary colic, jaundice, pruritus, elevated serum bilirubin, and abnormal cholestatic function tests [7]. Some may present with complications such as ascending cholangitis or acute pancreatitis [7]. Certain choledochal stones may be silent and are often discovered incidentally. Small, non-impacted stones may present intermittently due to a ‘ball-valve’ phenomenon [8].

Patients with large, multiple, or impacted stones usually present with clinical features of obstructive jaundice. There are various reports of CBD stone sizes, with the largest reported being 11.5 × 4 cm from India [9]. Another report describing a 7.5 × 4 cm stone is from the Republic of Korea [10].

The co-occurrence of gallbladder cancer with gallstones is well established, with a prevalence of approximately 80%–90% [2]. However the prevalence of gallbladder cancer in patients with CBD stones can be explained by their shared association with longstanding gallstone disease with gallstones.

Here, we report a patient with a giant CBD stone measuring 10.5 × 4.5 cm and weighing 150 g, associated with gallbladder cancer and normal cholestatic function tests, presenting with right upper quadrant abdominal pain of five months’ duration.

## Case summary

### Clinical findings

A 65-year-old Ethiopian female presented with right upper quadrant and epigastric abdominal pain of five months’ duration. The pain radiated to the back and was associated with decreased appetite, intermittent non-bilious vomiting, and significant unquantified weight loss. She had no history of jaundice, pruritus, or changes in stool or urine color. She was not taking any medications, including hormone replacement therapy, and had no known chronic medical illnesses.

On examination, her vital signs were within normal limits. She had pink conjunctiva and non-icteric sclera. Cardiovascular and respiratory examinations were unremarkable. Abdominal examination revealed a soft, non-tender abdomen without palpable masses.

### Laboratory finding

Blood panel results: hematocrit 33% (N: 40.1%–51%), Platelets 335 × 10^3^/uL (N: 163–337), liver enzymes: Alkaline phosphatase 143 (N: 35–104), and bilirubin levels: total 0.4 mg/dl and direct 0.14 mg/dl. All of which are in the normal range.

### Imaging

Ultrasound demonstrated asymmetric gallbladder wall thickening with multiple echogenic contents showing posterior acoustic shadowing. The CBD was markedly dilated (36 mm).

**Figure 1:**
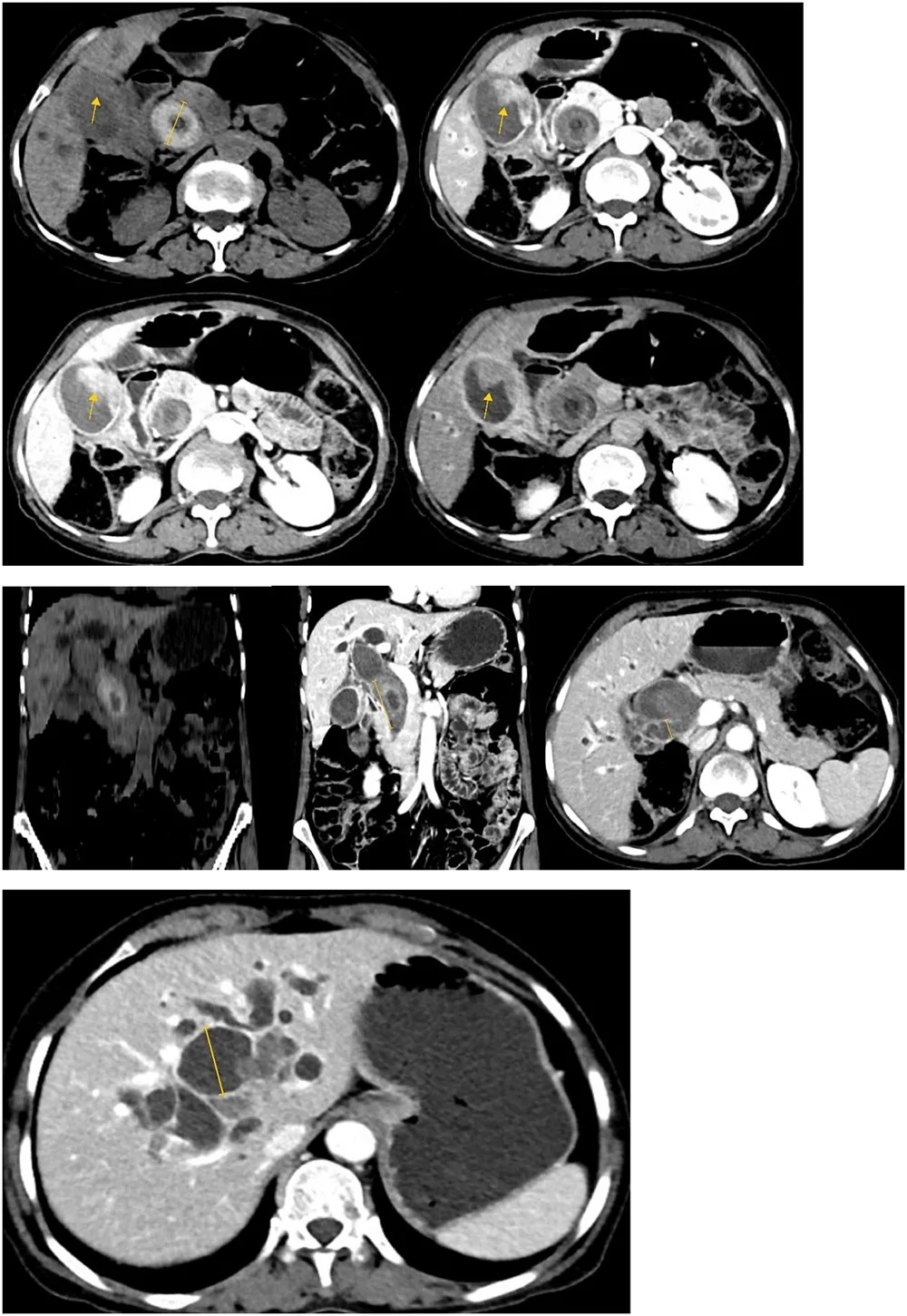
Contrast enhanced CT scan showing gall bladder wall thickening (short yellow arrow) and CBD stone (yellow caliper in the axial and coronal section).

Contrast-enhanced CT scan demonstrated a hyperdense stone filling the pancreatic portion of the CBD, asymmetric gallbladder wall thickening suggestive of malignancy, and marked dilation of the CBD, CHD, and intrahepatic ducts (Fig. 1). No metastasis was noted.

A preoperative diagnosis of locally advanced gallbladder cancer with massively dilated CBD secondary to choledocholithiasis was made.

### Operative findings

**Figure 2:**
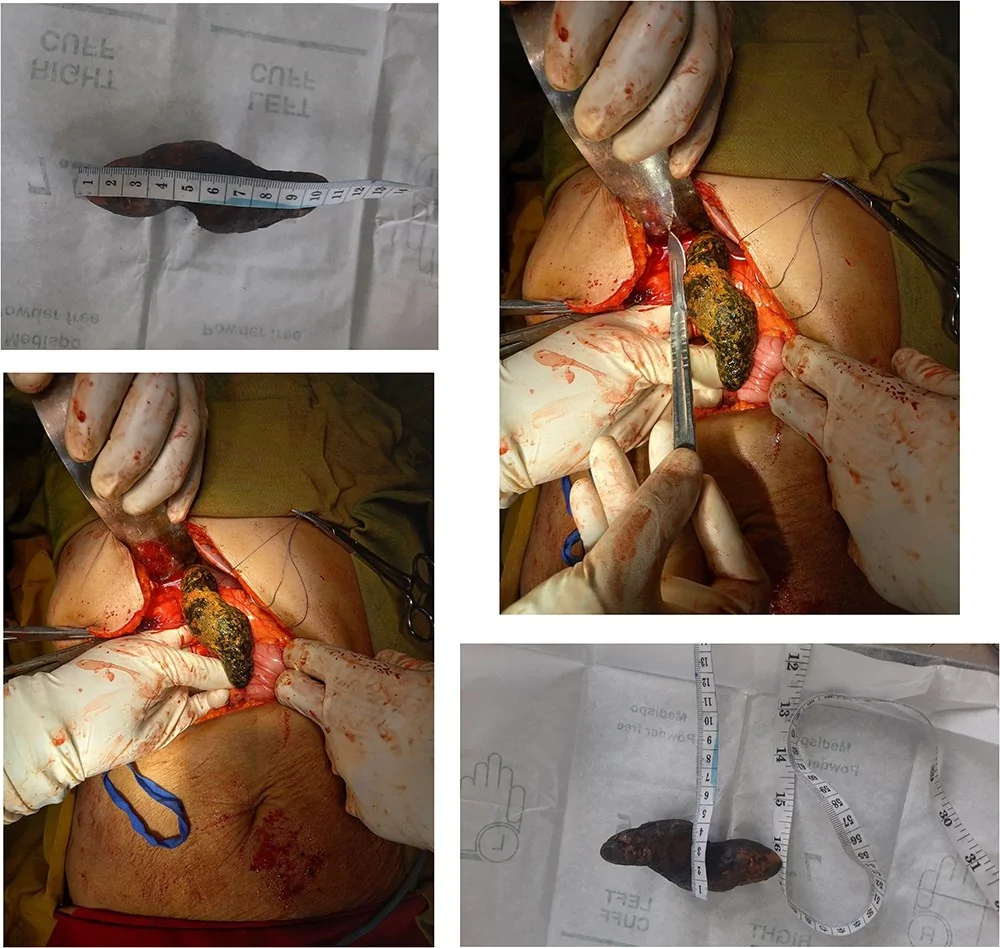
CBD stone shown intraoperatively and after removal.

Intraoperatively, there were adhesions between the omentum, transverse colon, and gallbladder fundus. A palpable mass over the body of the gallbladder invaded the proximal transverse colon. The CBD was markedly dilated and uniformly filled with a large single stone occupying its entire length and extending into the CHD (Fig. 2). No regional or distant lymphadenopathy was identified.

The patient underwent extended cholecystectomy with resection of liver segment IVB and part of segment V, en-bloc wedge resection of the involved colon, CBD exploration via longitudinal choledochotomy, and extraction of a 10.5 × 4 cm stone weighing 150 g. Choledochoduodenostomy was performed because of its advantage of no tension and the distal location of the pathology and to avoid relatively more morbid procedure.

Estimated blood loss was 600 mL, and one unit of packed red blood cells was transfused intraoperatively.

### Postoperative course

On postoperative day four, the patient developed a superficial surgical site infection and was treated with intravenous ceftriaxone for one week and appropriate wound care.

Histopathology confirmed adenocarcinoma of the gallbladder with negative margins.

After two weeks of inpatient care, she was clinically stable, tolerating a regular diet, and was discharged home. Over the following 2 years, she has been well with no detectable tumor recurrence. No adjuvant chemotherapy was required.

## Discussion

It is common to encounter giant stones in the gallbladder but uncommon in the common bile duct [11]. There is variation in the cut-off size defining a ‘giant’ CBD stone, with most reports defining stones greater than 5 cm as giant [12–14]. Only a few giant CBD stones have been reported, most from Asian countries where primary CBD stones are more common [11].

To the best of the authors’ knowledge, there are no prior reports from Africa describing a CBD stone of this size. In our case, the stone measured 10.5 × 4.5 cm, making it one of the largest reported in Ethiopia and Africa.

Most patients with giant CBD stones present with obstructive jaundice and abnormal cholestatic function tests [10, 15]. Akshay B. *et al*. reported that only 6 of 12 patients with giant CBD stones had jaundice [11]. Our patient had no jaundice, pruritus, or changes in stool or urine color, and her cholestatic parameters were within normal limits.

The coexistence of gallbladder cancer with gallstones is well established, with a reported prevalence of approximately 80%–90% [2]. In this case, the presence of a giant CBD stone may reflect longstanding gallstone disease, as CBD stones commonly result from migration of gallstones into the common bile duct. Prolonged stone impaction may cause biliary obstruction, bile stasis, and chronic inflammation, which may contribute to gallbladder carcinogenesis. Although the coexistence of a giant CBD stone and gallbladder cancer may therefore suggest a possible link through chronic biliary inflammation, this association remains speculative. Its implication is that a giant CBD stone may serve as a marker of longstanding biliary stone disease and chronic inflammatory exposure in patients with gallbladder cancer; however, further studies are needed to determine whether this represents a true pathogenic association or a coincidental coexistence.

## Conclusion

Giant CBD stones are rare, especially in Africa. They typically present with abnormal cholestatic function tests and jaundice. However, this case demonstrates that even a massive CBD stone may present with normal laboratory parameters.

The coexistence of gallbladder cancer and a giant CBD stone represents longstanding gallstone disease and chronic inflammatory changes that contribute to the development of gallbladder cancer.

## Data Availability

Data are available at Tikur Anbessa Specialized Hospital upon reasonable request.

## Abbreviations

CBD, Common bile duct; GBC, Gallbladder cancer; CBDE, Common bile duct exploration
